# A Potentially Misleading Hepatocellular Carcinoma

**DOI:** 10.3390/medicina57080850

**Published:** 2021-08-20

**Authors:** Ottavia De Simoni, Andrea Barina, Mario Gruppo, Antonio Scapinello, Vasileios Mourmouras, Pierluigi Pilati, Boris Franzato

**Affiliations:** 1 Surgical Oncology of Digestive Tract Unit, Veneto Institute of Oncology, IOV-IRCCS, Castelfranco Veneto (TV), Via Dei Carpani 16/Z, 35128 Padova, Italy; ottavia.desimoni@iov.veneto.it (O.D.S.); mario.gruppo@iov.veneto.it (M.G.); pierluigi.pilati@iov.veneto.it (P.P.); boris.franzato@iov.veneto.it (B.F.); 2 Anatomy and Histology Unit, Veneto Institute of Oncology, IOV-IRCCS, Castelfranco Veneto (TV), Via Dei Carpani 16/Z, 35128 Padova, Italy; antonio.scapinello@iov.veneto.it (A.S.); vasileios.mourmouras@iov.veneto.it (V.M.)

**Keywords:** hepatocellular carcinoma, accessory liver lobe, case report

## Abstract

Hepatocellular carcinoma (HCC) typically presents in patients with a chronic liver disease and rarely develops in healthy liver, especially within an accessory liver lobe. We present a case of a healthy 64-years-old woman who showed a serum alpha-fetoprotein (AFP) value of 226.3 µg/mL during a screening blood test. Past medical history was negative for chronic liver disease or cirrhosis. Intraoperative finding was an ovaloid mass connected with the second hepatic segment by a thin pedicle of hepatic tissue. Lesion was safely resected by laparoscopic approach. Histopathology analysis showed a trabecular hepatocellular carcinoma. After a 6-month follow up, there was no evidence of recurrent disease. This case report showed how serum AFP remains a highly sensitive marker, although the presentation of HCC was unusual. To our knowledge, this is the second case reported in the literature.

## 1. Introduction

Hepatocellular carcinoma (HCC) is the most common primary liver cancer and accounts for the fifth most common cancer worldwide and the third most common cause of cancer mortality [1].

HCC largely occurs within an established background of chronic liver disease and cirrhosis (70–90% of all detected HCC cases). Hepatocarcinogenesis is linked tightly to chronic liver damage but rarely develops in healthy liver during normal aging [2].

Accessory liver lobes (ALL) are defined as morphologic variations of the liver due to excessive development of hepatic tissue [3,4]. ALL is defined as a supernumerary liver lobe, composed of normal liver parenchyma attached to the liver by a bridge of hepatic tissue, a mesentery or by a stalk [5]. Riedel’s lobe is the most well-known of accessory liver lobes, corresponding to hypertrophy of segments V and VI [6,7]. Conversely, ectopic liver lobes have no anatomical continuity with the normal liver [8].

ALL is usually asymptomatic. The presence of an ALL is often discovered occasionally and revealed by imaging. When symptomatic, ALL presents typically with abdominal pain due to its torsion, especially when the lobe is pedunculated [9]. To our knowledge, only one case shows neoplastic degeneration within ALL [10].

Herein, we present a case of HCC arising from an asymptomatic ALL.

## 2. Case Report

**Figure 1:**
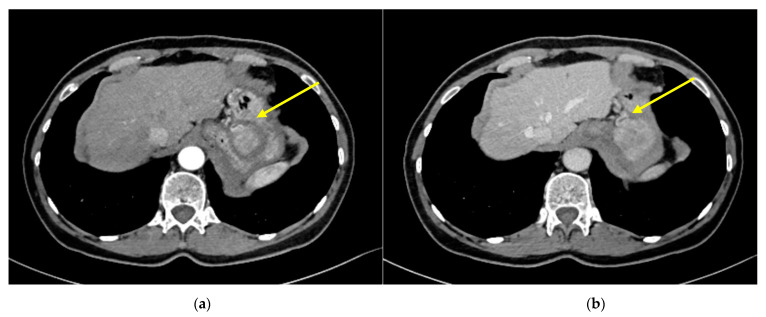
Axial contrast-enhanced computed-tomography (CT)-scan images: (**a**) arterial phase; (**b**) portal phase. Arrow: ovaloid-shaped solid mass.

A 64-years-old woman in regular follow up after a microscopic transphenoidal surgery for pituitary adenoma was referred to our Unit due to an elevation of serum alpha-fetoprotein (AFP). Laboratory tests revealed serum AFP value of 226.3 µg/mL (normal range: 0–7.4 µg/mL). Serologic markers were negative for hepatitis B or C viruses infection. Past medical history was negative for chronic hepatitis, alcohol abuse, or liver cirrhosis as well as family history for hepatocarcinoma. Patient was asymptomatic and physical examination showed normal findings, especially no evidence of hepatomegaly or weight loss. Contrast enhanced computed-tomography (CT) scan of the abdomen showed an ovaloid-shaped solid mass in the epigastrium very closed to the fundus of the stomach (Figure 1).

Esophagus-gastroduodenoscopy revealed an imprint of the gastric mucosa of the fundus, compatible with gastro-intestinal stroma tumor (GIST). Furthermore, an ultrasound endoscopy was performed showing an oval-shaped mass of 40 mm by 20 mm compatible with gastric leiomyoma.

**Figure 2:**
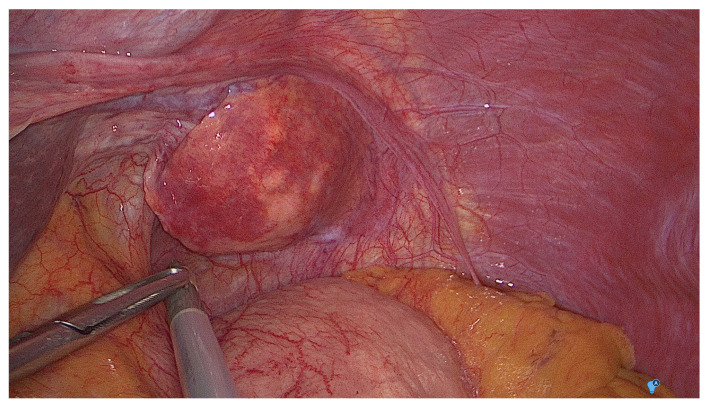
Intraoperative findings. Between the anterior gastric wall and the diaphragm muscle, the ovaloid mass is clearly visible within an accessory liver lobe. LHL: left hepatic lobe; AGW: anterior gastric wall; HCC: hepatocellular carcinoma.

**Figure 3:**
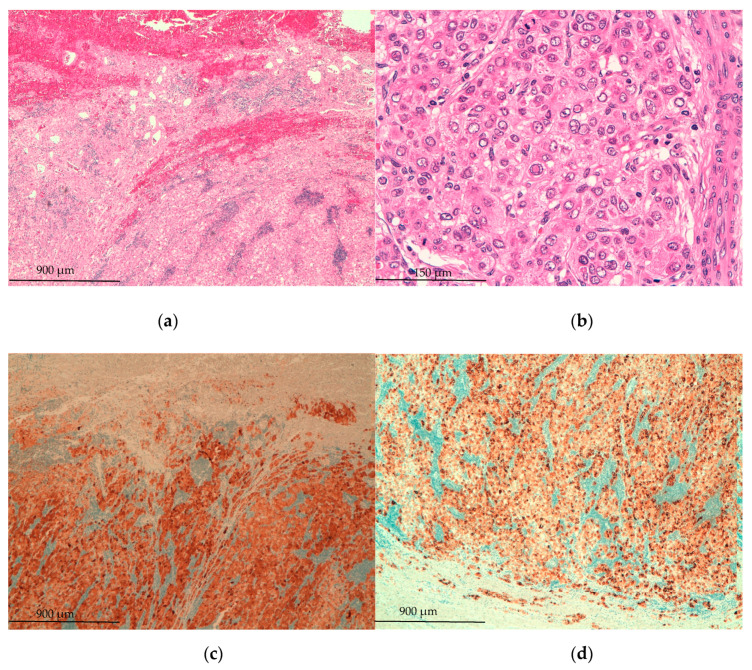

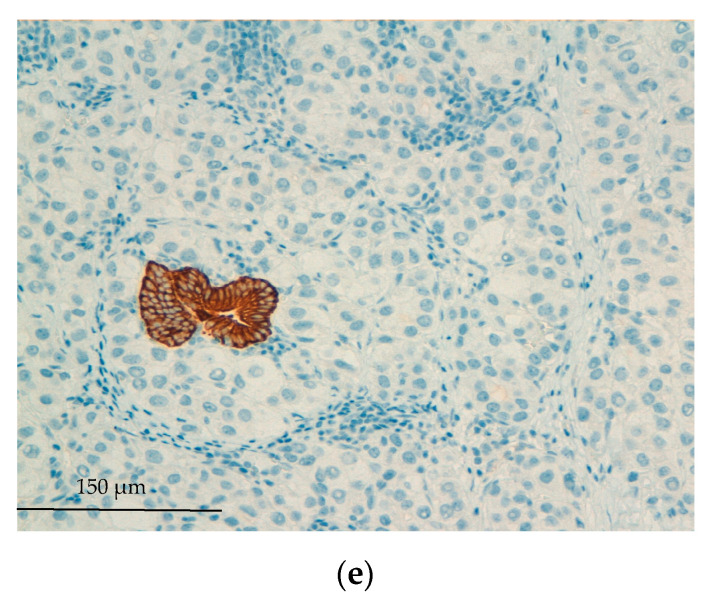
Histopathology images: (**a**) H&E stain, zoom 5×. Solid and trabecular epithelioid neoplasm with peripheral hemorrhage and dilated small vessels corresponding to peduncle. Note the presence of perivascular small lymphocytes aggregates; (**b**) H&E stain, zoom 30×. Solid area. The cells show atypical nuclei with frequent nuclear pseudoinclusions; (**c**) arginasea1 immunohistochemistry stain, zoom 5×: strong and diffuse cytoplasmic positivity in tumor cells that confirm the hepatocellular differentiation of neoplastic cells; (**d**) glypican3 immunohistochemistry stain, zoom 5×: strong and diffuse membranous positivity in tumor cells that support the malignant nature of hepatocellular proliferation. (**e**) CK19 immunohistochemistry stain, zoom 30×: absence of reactivity in the neoplastic cells, with positive internal control in a non-neoplastic ductal structure. H&E: hematoxylin and eosin.

After these preoperative investigations, the patient went to the operation room and laparoscopy was performed. Intraoperative finding was an ovaloid mass located in the left hypochondrium connected with the second hepatic segment by a thin pedicle of hepatic tissue. (Figure 2) The mass was resected and histopathology analysis showed a trabecular HCC, moderately differentiated with vascular invasion. Immunohistochemistry analysis was as followed: Arginase+, Glypican3+, CK19- (Figure 3). Postoperative period was uneventful and patient was discharged on postoperative day 2.

The value of serum AFP was 219.6 UI/mL immediately after surgery and 93.9 UI/mL on postoperative day 7, respectively. The value of serum AFP returned to normal values gradually. Oncologic consultation did not recommend adjuvant chemotherapy.

## 3. Discussion

Primary liver cancers, of which majority are HCCs, are now the fifth and ninth most frequent cancer in men and women, respectively [11]. HCC development depends upon a multitude of factors including etiology and severity of underlying liver disease, host, lifestyle, behavioral and environmental risk factors [12]. HCC arises almost exclusively within the setting of chronic liver disease and the patient has underlying cirrhosis in up to 90% of cases [13,14].

Pathological alterations of liver parenchyma and hepatic perfusion, as seen in fibrotic changes by cirrhosis, lead to a decrease in hepatocyte function and a growth in transhepatic perfusion resistance, resulting in portal hypertension and significantly increase in mortality and morbidity [15].

The occurrence of accessory liver lobe is caused by an error in the formation of the endodermal caudal foregut during the third gestational week and segmentation of the hepatic bud [16].

An accessory liver lobe is a rare congenital anomaly, usually asymptomatic. An observational laparoscopic study revealed that the incidence of intraoperative accessory liver lobe and ectopic liver was 0.7% [17]. Such ectopic liver tissue, with its own mesentery, lacks the anatomical fixation by the liver ligaments rendering it susceptible to rotation or even torsion, especially when huge in size.

The association between accessory liver lobes and pedunculated tumors has not been clearly shown. Pathophysiology of HCC is assumed to be the same regardless of its growth on the accessory lobe and its association with liver cirrhosis, although no clear evidence is currently available [18]. Some authors [19,20] suggest that degeneration of an ALL may be due to a compromised vascular supply or a compromised biliary drainage. Whether the patient was affected by HCC within ALL or a pedunculated HCC (pHCC) was unclear until surgery. PHCC is a rare form of cancer, which protrudes from the liver with or without a pedicle [21]. Differential diagnosis with ALL is challenging, since the exophytic growth of the tumor lies beyond the confines of the liver. An uncommon origin to consider may be a degenerated ectopic hepatic adenoma.

Intraoperative findings were more consistent with a case of ALL than pHCC and histopathology analysis confirmed.

The literature does not give any indication concerning prognosis of HCC within ALL as compared to intrahepatic parenchymal tumors. Of the three largest series in the literature comparing pedunculated to non-pedunculated hepatocellular carcinomas, evidence is poor to determine whether the prognosis of resected pedunculated tumors is comparable with non-pedunculated tumors [21,22,23].

Treatment of HCC would not differ whether the tumor is located in ALL. When feasible, surgical resection remains mandatory.

This case report shows that HCC may grow within ALL and could often remain asymptomatic or can be misdiagnosed. Evaluation of serum neoplastic markers is useful to direct diagnosis. Laparoscopy remains mandatory both in diagnosis and surgical treatment.

## 4. Conclusions

The development of HCC may occur within an ALL and is an extremely rare presentation. The diagnosis is mostly accidental and the treatment is surgical resection. Serum AFP remains an extremely sensitive marker.

## Data Availability

Data are available on patient’s medical record. I choose to exclude this statement.

## References

[1] Parkin D.M. (2001). Global cancer statistics in the year 2000. Lancet Oncol..

[2] El-Serag H.B., Rudolph K.L. (2007). Hepatocellular Carcinoma: Epidemiology and Molecular Carcinogenesis. Gastroenterology.

[3] Champetier J., Yver R., Letoublon C., Vigneau B. (1985). A general review of anomalies of hepatic morphology and their clinical implications. Anat Clin..

[4] Khan A.M., Hundal R., Manzoor K., Dhuper S., Korsten M.A. (2006). Accessory liver lobes: A diagnostic and therapeutic challenge of their torsions. Scand. J. Gastroenterol..

[5] Gaber M. (1980). Accessory liver containing metastatic tumour. Virchows Arch. A Pathol. Anat. Histol..

[6] Riedel I. (1888). Uber den zungenformigen Forsarz des rechten Leberlappens und seine pathognostische Bedeutung fur die Erkrankung der Gallenblase nebst Bemerkungen uber Gallenstein Operationen. Berl. Klin. Wochenschr..

[7] Gillard J.H., Patel M.C., Abrahams P.H., Dixon A.K. (1963). Riedel’s lobe of the liver. Am. J. Surg..

[8] Morris M.W., Helling T.S., Creswell L.L., Jordan B., Mitchell M.E. (2012). Ectopic liver masquerading as a floating intracaval mass. J. Vasc. Surg..

[9] Kostov D.V., Kobakov G.L. (2011). Accessory hepatic lobe. Surg. Radiol. Anat..

[10] Wang X., Zhang Q., Xu K. (2019). Hepatocellular carcinoma arising from left accessory liver lobe supplied by the branch of left hepatic artery. A case report. Medicine.

[11] Ferlay J., Soerjomataram I., Ervik M., Dikshit R., Eser S., Mathers C., Rebelo M., Parkin D.M., Forman D., Bray F. (2015). Cancer incidence and mortality worldwide: Sources, methods and major patterns in GLOBOCAN 2012. Int. J. Cancer.

[12] Wallace M.C., Preen D., Jeffrey G.P., Adams L.A. (2015). The evolving epidemiology of hepatocellular carcinoma: A global perspective. Expert Rev. Gastroenterol. Hepatol..

[13] Chiesa R., Donato F., Tagger A., Favret M., Ribero M.L., Nardi G., Gelatti U., Bucella E., Tomasi E., Portolani N. (2000). Etiology of hepatocellular carcinoma in Italian patients with and without cirrhosis. Cancer Epidemiol. Biomark. Prev..

[14] Simonetti R.G., Cammà C., Fiorello F., Politi F., D’Amico G., Pagliaro L. (1991). Hepatocellular carcinoma. A worldwide problem and the major risk factors. Dig. Dis. Sci..

[15] Schuppan D., Afdhal N.H. (2008). Liver cirrhosis. Lancet.

[16] Carrabetta S., Piombo A., Podestà R., Auriati L. (2009). Images in surgery: Torsion and infarction accessory liver lobe in young man. Surgery.

[17] Sato S., Watanabe M., Nagasawa S., Niigaki M., Sakai S., Akagi S. (1998). Laparoscopic observations of congenital anomalies of the liver. Gastrointest. Endosc..

[18] Yeh C.N., Lee W.C., Jeng L.B., Chen M.F. (2002). Pedunculated hepatocellular carcinoma: Clinicopathologic study of 18 surgically resected cases. World J. Surg..

[19] Arakawa M., Kimura Y., Sakata K., Kubo Y., Fukushima T., Okuda K. (1999). Propensity of ectopic liver to hepatocarcinogenesis: Case reports and a review of the literature. Hepatology.

[20] Caygill C., Gatenby P. (2004). Ectopic liver and hepatocarcinogenesis. Eur. J. Gastroenterol. Hepatol..

[21] Karatzas T., Smirnis A., Dimitroulis D., Patsouras D., Evaggelou K., Kykalos S., Kouraklis G. (2011). Giant pedunculated hepatocellular carcinoma with hemangioma mimicking intestinal obstruction. BMC Gastroenterol..

[22] Horie Y., Shigoku A., Tanaka H., Tomie Y., Maeda N., Hoshino U., Koda M., Shiota G., Yamamoto T., Kato S. (1999). Prognosis for pedunculated hepatocellular carcinoma. Oncology.

[23] Woodall C.E., Scoggins C.R., Loehle J., Ravindra K.V., McMasters K.M., Martin R.C. (2007). Hepatic imaging characteristics predict overall survival in hepatocellular carcinoma. Ann. Surg. Oncol..

